# Experiences of patients with rheumatoid arthritis during and after COVID‐19‐induced quarantine in terms of physical activity and health status: A qualitative study

**DOI:** 10.1111/jonm.13784

**Published:** 2022-09-20

**Authors:** Laura Ramos‐Petersen, Jonatan García‐Campos, George Banwell, Ana Belén Ortega‐Ávila, Gabriel Gijon‐Nogueron, Andrés Reinoso‐Cobo

**Affiliations:** ^1^ Department of Podiatry, Faculty of Health Sciences Universidad Católica San Antonio de Murcia, Campus de Los Jerónimos Murcia Spain; ^2^ Research Team PODUMA University of Malaga Malaga Spain; ^3^ Department of Behavioral Sciences and Health, Faculty of Medicine Miguel Hernandez University Alicante Spain; ^4^ Institute for Health and Biomedical Research (ISABIAL) Alicante Spain; ^5^ Department of Nursing and Podiatry, Faculty of Health Sciences University of Malaga Malaga Spain; ^6^ IBIMA Malaga Spain

**Keywords:** COVID‐19, exercise, health status, rheumatoid arthritis

## Abstract

**Aim:**

The aim of this study was to explore experiences of people with rheumatoid arthritis during and after COVID‐19‐induced quarantine in terms of physical activity and health status.

**Background:**

Rheumatoid arthritis affects multiple facets of the person, both physically and psychologically. Physical activity is considered a safe and effective intervention to improve symptoms and systemic manifestations of rheumatoid arthritis. In the context of the COVID‐19, countries like Spain were forced to impose restrictions on mobility, prohibiting outings even to perform physical activity.

**Methods:**

Structured interviews were conducted and developed using the Tampa Scale for Kinesiophobia‐11 questionnaire. Data were analysed using a six‐step thematic analysis.

**Results:**

The results make it clear that even though the patients declared that physical activity is essential for them to deal with their disease, most of the participants affirmed that they significatively reduced their levels of physical activity during the pandemic.

**Conclusions:**

Physical activity should be promoted in people, even in difficult times, to improve disease outcomes, well‐being and mental health.

**Implications for Nursing Management:**

Knowing the experiences of these patients enables nursing managers to develop interventions that ensure the delivery of comprehensive nursing care regarding physical activity and health status, in future situations like this pandemic.

## 1 BACKGROUND

Rheumatoid arthritis is an inflammatory, autoimmune and chronic disease that causes: destruction of synovial joints, local inflammation and joint pain, with associated comorbidity, disability and premature mortality (Aletaha et al., 2010; McInnes & Schett, 2017; Smolen et al., 2016). In Spain, a prevalence of 0.82% (95% CI: 0.59–1.15) was estimated (Silva‐Fernández et al., 2020).

Rheumatoid arthritis affects multiple facets of the person, both physically and psychologically; gait adaptations have been identified in these patients (Carroll et al., 2015), as well as driving difficulties and the use of driving adaptations (Zhou et al., 2021) and high rates of work disability (Sokka et al., 2010). In some patients, there appears to be a relationship between disease activity and fatigue (Versteeg et al., 2022). Depression is also highly prevalent in this population (Matcham et al., 2013), as well as anxiety (Katchamart et al., 2020) and increased cardiovascular risk (Restivo et al., 2022), finding that rheumatoid arthritis has a significant impact on health‐related quality of life (Matcham et al., 2014). In contrast, physical activity and exercise are considered safe and effective interventions to improve symptoms and systemic manifestations of rheumatoid arthritis, improving cardiovascular health (Metsios & Kitas, 2018), fatigue (Durcan et al., 2014; Katz et al., 2018; Rongen‐van Dartel et al., 2015), sleep (Durcan et al., 2014), depressive symptoms (Kelley et al., 2015), pain (Durcan et al., 2014) and cognitive function (Azeez et al., 2020).

In response to the declaration of a global pandemic by coronavirus disease 2019 (COVID‐19) decreed by the World Health Organization (WHO) in March 2020 (World Health Organization, n.d.), many countries were forced to impose restrictions on the mobility of citizens. In the case of Spain, a state of alarm was declared throughout the national territory where the freedom of movement of people was limited during its validity, prohibiting outings even to carry out physical activity (RD 463/2020, de 14 de marzo, n.d.). This confinement drastically affected the reduction in the practice of physical activity and the increase in sedentary lifestyle in the general population (Ammar et al., 2020; López‐Bueno et al., 2020; Wunsch et al., 2022), and in people with rheumatoid arthritis in particular (Balchin et al., 2022; Lévy‐Weil et al., 2021). In the case of people with rheumatoid arthritis, the decrease in sports and physical activity has been correlated with an increase in relapses (Ammar et al., 2020), an increase in body weight (World Health Organization, n.d.) and a worse quality of life (Lévy‐Weil et al., 2021), while performing physical activity, especially light physical activity and walking, is positively associated with less mental and physical fatigue and better psychological well‐being (Brady et al., 2021), and the European Alliance of Associations for Rheumatology (EULAR) in 2012 determined that the role of the nurse in the management of chronic inflammatory arthritis (CIA) is essential. These recommendations provide a basis for emphasizing and optimizing rheumatology nursing care in order to contribute to a more standardized level of professional nursing across Europe. The following is the 10th recommendation by EULAR: ‘Nurses should carry out interventions and monitoring as part of comprehensive disease management in order to achieve cost savings’ (van Eijk‐Hustings et al., 2012).

Although few studies have raised this problem from a qualitative analysis, this analysis raises precisely in this population a very adequate research base due to the need for communication and not only raw quantitative data. Therefore, the objective of this study was to explore experiences of people with rheumatoid arthritis during and after COVID‐19‐induced quarantine in terms of physical activity and health status.

## 2 METHODS

This study adopted a qualitative approach to explore experiences of people with rheumatoid arthritis during and after COVID‐19‐induced quarantine in terms of physical activity and health status.

### 2.1 Ethical considerations

This qualitative study received ethical approval from the committee of Portal de Ética de la Investigación Biomédica de Andalucía (PEIBA) (SPAR‐001), which was authorized and extended to a longitudinal study ARC0001. This study was carried out in full accordance with the provisions of the Declaration of Helsinki regarding ethical principles for medical research involving human subjects and was approved by the Ethics Committee.

### 2.2 Participants

A convenience sample of people with rheumatoid arthritis was recruited from the Hospital Virgen de las Nieves, Granada (Spain).

Inclusion criteria were that the participant was consenting, participants aged 18 or over, satisfied the 2010 Rheumatoid Arthritis Classification Criteria (approved by the American College of Rheumatology and the European League Against Rheumatism) (Aletaha et al., 2010) and the ability to express themselves in Spanish in order to understand and respond to the questions.

Participants were excluded if they presented dementia, other rheumatic diseases, diabetes, cardiovascular diseases, previous lower limb surgery or amputation.

They all agreed to take part in the study and provided informed and written consent. Interviews took place from January to March 2021.

Eligible participants were contacted by phone to ascertain willingness to participate in the qualitative study.

### 2.3 Data collection

Data were collected using a structured interview. Multiple interviews were carried out to achieve data saturation, meaning that the themes are repeated in some interviews. The following quantitative data were also collected prior to the start of the study:

- Demographic information such as gender and date of birth.
- Levels of pain, both general and foot specific measured with the Visual Analogue Scale (VAS) (Sendlbeck et al., 2015).
- Fear of movement, health status and confidence level in performing daily activities were measured using the Tampa Scale for Kinesiophobia (TSK11) (Gómez‐Pérez et al., 2011).

The TSK‐11 is an 11‐item self‐report checklist using a 4‐point Likert scale, being 1 *strongly disagree* and 14 *strongly agree*. Low scores mean no kinesiophobia, and high scores indicate there is kinesiophobia.

The interviews were conducted by phone, and they were recorded using a digital voice recorder. Field notes supplemented the data. The interviews were carried out by a researcher who had experience with patients with rheumatoid arthritis from both a clinical and research context of the interview, and participants were made aware that the researcher was also a podiatrist and nurse from the rheumatology department of the hospital (Richards & Emslie, 2000). A code was assigned for each patient, so they were anonymized or pseudonymized. There was only one person from the research team (data manager) who knew the relationship between the assigned code and the patient's clinical history, leading to a blind code. That member accessed the files with a password and was not part of the data collection process, to not interfere. All the reports are stored in the computer system used by the hospital, so the people who access that system must have permission from management, as they have passwords and individual credentials.

Informed consent forms were printed to be signed by the participants. All these signed consents, together with the evaluation and data collection sheets, are archived in an office for 5 years, to which only authorized members have access. After 5 years, those folders will go to a warehouse belonging to the hospital's rheumatology department, where all the documents related to studies that have been carried out are kept, and they will remain under lock and key for 15 years. Later, they will be destroyed.

Data analyses were conducted by two researchers.

The questions for the structured interview were developed using the questionnaire TSK‐11 as a guide and from a review of the literature on COVID‐19‐induced quarantine in terms of physical activity and health status. The research question that was addressed in this qualitative study was as follows: *How were the physical activity levels and health status of people with rheumatoid arthritis during and after COVID‐19‐induced quarantine?* Therefore, the following questions were asked to the participants:

- Did you test positive for COVID‐19?
- Has your level of physical activity been influenced by being positive for COVID‐19 or by the COVID‐19‐induced quarantine?
- Did you suffer worse symptoms, pain or flares?
- Did the COVID‐19‐induced quarantine influence your level of physical activity?
- During the COVID‐19‐induced quarantine, did you have to make a change in your level of physical activity due to fear?
- Did the level of physical activity decrease, increase or was it the same?
- Have you felt motivated to increase your level of physical activity?
- Have you had to carry out any extra activity that requires a greater expenditure of energy? (e.g. taking care of the grandchildren)
- Have you felt more vulnerable to testing positive for COVID‐19 due to your rheumatoid arthritis?
- Do you think that rheumatoid arthritis treatments could protect you against COVID‐19?

No repeat interviews were conducted, and transcripts were not returned to participants for comment.

### 2.4 Data analysis

The six‐step thematic analysis framework from Braun and Clarke (2006) was followed for the data analysis. One researcher undertook a line‐by‐line analysis of the transcribed experiences of each participant.

From that work, another researcher (LRP) read the field notes and all the transcripts, and codes were developed. Initial codes were then collated to gather data into themes. Particular attention was paid to both the frequency of emerging codes and their importance for multiple participants. Coded extracts were reviewed within their themes and afterward defined and named. MAXQDA qualitative data analysis software was used to facilitate coding and analysis. The findings were then scrutinized by the co‐authors.

The quantitative information obtained is reported as median and interquartile range (IQR) due to the non‐normal distribution of the variables and as mean and standard deviation (SD) due to the normal distribution of the variables. Normality of the distribution was examined using the Shapiro–Wilk test. All statistical analyses were conducted using SPSS V.24.0 statistical software.

## 3 RESULTS

A total of 23 interviews were analysed thematically. Eighty of the participants were female, and five were male, aged between 44 and 79 years old (mean 59.6 years old). The range of rheumatoid arthritis disease duration was between four and 40 years (mean 19.04 years). In terms of drugs for managing rheumatoid arthritis, 91% of participants were receiving biologics, 52% methotrexate, and 47% corticosteroids. The details of the participants are available in Table 1.

**TABLE 1:** Characteristics of the participants

	Mean (SD)/median [IQR]
RA duration	17[12–23]
Age	60[53–64]
Weight	68[61–78]
Height	168[160–170]
General VAS score	5.7(3.4)
Feet VAS score	5(3.9)
TSK11 score	31[28–36]
DAS 28	3.06[2–4.3]
SDAI	12.1[6.1–19.6]
ESR	21.7(18.9)
CRP	1.45(1.76)

Abbreviations: CRP, C‐reactive protein; DAS, disease activity score; ESR, erythrocyte sedimentation rate; RA, rheumatoid arthritis; SDAI, Simplified Disease Activity Index; TSK‐11, Tampa Scale for Kinesiophobia; VAS, Visual Analogue Scale.

Seventy‐seven codes were identified, which were then organized into four themes. The resulting themes were agreed by the researcher and co‐authors to enhance the validity of the data:

- Physical activity detriment.
- Health detriment.
- Social implications of COVID‐19 pandemic.
- Vulnerability of testing positive for COVID‐19 due to rheumatoid arthritis.

### 3.1 Theme 1: Physical activity detriment

Some participants defend the importance of maintaining a good level of physical activity when suffering from rheumatoid arthritis (especially pilates, swimming and walking), emphasizing that they feel better when they do sports. Even so, only a minority of the participants have not reduced their level of physical activity, stating that they feel better, both physically and psychologically, even though they have been forced to change their activity due to confinement:

I am completely sure that physical activity helps me with my illness, in confinement my ankles swelled and turned black. I didn't go out for a walk and every day I have to do quite a few kilometres to feel good. (COVAR00010)

I was going to a gym, and I had to stop going. I have had to change activities. (COVAR0004)

Even so, the vast majority have declared a decrease in physical activity due to the pandemic. This is due both to restrictions, fear or lack of social interaction, not feeling in the mood to play sports alone:

Before I used to walk a lot, but now I'm afraid to go out, it's hard for me, I get very nervous. (COVAR00010)

I even bought a stationary bike, some dumbbells and equipment to exercise at home, but it's not the same, in the end I get lazy, and I don't do it. (COVAR00011)

I did not dare to go out, I was afraid. Also, it is not the same to go for a walk alone. In general, I have felt very lonely. (COVAR0002)

I have had a more sedentary phase. (COVAR0008)

In some cases, physical activity has not been reduced because it was already minimal before the pandemic, due to the pathology and the pain they suffer:

It hasn't influenced me because I didn't do anything extraordinary either. (COVAR0006)

In general, I am not very athletic. I do not like to exercise. (COVAR0009)

Once the confinement ended, some participants declared that they tried to increase the level of physical activity, but because it had decreased, they felt that the difficulty had increased, becoming impossible in some cases:

We are starting to go for a walk, but it has to be little by little, because right now I feel that my joints are tight. I do what I can, but very limited. Compared to before, it is almost nothing, and I try it because I think that by exercising, I feel better. (COVAR0013)

Before the pandemic I used to walk a lot and now it's hard for me, I'm suffocating. (COVAR0011)

Everything is harder for me now, it's hard for me to climb the stairs, I can't walk like I used to, I have a hard time. Now I don't do half of the things I could do before, I feel very slow. (COVAR0002)

After being stuck at home for so long without being able to go out, it shows that now everything is much harder for me than before. (COVAR0021)

### 3.2 Theme 2: Health detriment

In the same way that the vast majority declared a decrease in physical activity due to the pandemic, in general, the participants declared that the level of pain had increased compared to their pre‐pandemic state:

That physical inactivity affected me a lot, it meant I had more pain and swollen ankles. Also, I ate more and gained weight, having more stress, I guess that would be all …. (COVAR0010)

The pain has increased and also the outbreaks, my knees and lower back hurt more, everything in general, it hurts much more. My legs also hurt a lot. (COVAR0002)

The symptoms of my disease were increased, I had a lot of pain. My feet hurt a lot. (COVAR0003)

The pain keeps increasing if I try to do more activities. (COVAR0005)

In addition, several participants stated that they did not feel that their body had the same capacities as before, suffering from a worsening in their health and a decrease in their capacities:

I noticed how my legs did not respond to me as usual. (COVAR0007)

I felt more rigid, especially in the area of the hips and legs, from that routine from bed to chair and repeating that every day. (COVAR0009)

Now I feel suffocated. I also notice it when I go to the supermarket and bend down to pick up a product, that it is difficult for me to get up and I consider myself a young person. I see that I don't have the skills I had before, even doing housework, I don't have the strength I used to and many afternoons I have to sit down. (COVAR0011)

I had to go up a hill, but I couldn't, and I used to go up and down with no problem. (COVAR0017)

Now I spend more time locked up and I do little, I notice that it is more difficult for me, so I leave what I am doing halfway, and I sit or lie down. My legs have gotten worse. (COVAR0018)

As their heath had worsened, they could not perform physical activity, or vice versa, as they had become physically inactive their health had worsened.

I don't know if the pain is due to my illness or because I've spent so much time doing nothing. (COVAR0013)

I don't know if it's because I'm less active, but I find myself with less agility. I have lost agility and strength. (COVAR0011)

After the months of confinement, I couldn't even go out, I'm much worse now. So much time locked up, without being able to do anything and when we were able to get out I couldn't. (COVAR0019)

The disease is showing symptoms that are a bit out of control, before it affected my arms, elbows and shoulders more, and now my knees and feet are awful, but I don't know if it's a consequence of being inactive. (COVAR0014)

By not moving, it seemed that my joints hurt more, they bothered me more, as if they were stiffer in my feet and hands. It is as if the joints were asleep and in a lot of pain. (COVAR0021)

### 3.3 Theme 3: Feelings and social implications of COVID‐19 pandemic

The pandemic awakens many feelings, such as missing practicing physical activity with a group of friends:

I really missed the walks, because I didn't go alone, I went with other people. I've had a bad time locked up. (COVAR0001)

Before I was walking, and it was good for me to relate and distract myself. But now, I'm more depressed, because I can't go out and not see my friends. It was horrible not being able to go out at all and I had a terrible time. I felt sadder and more tired. (COVAR0016)

The pandemic has caused most of the participants to be afraid and are still afraid. In some cases, there is even talk of depression. Sometimes, this fear is due to considering themselves more vulnerable due to their illness:

I'm going to stay locked up at home and go out just enough, because I'm very scared. (COVAR0016)

With the fear that I have, I don't even want to go out on the street. It has changed my behaviour. (COVAR0017)

What I have had has been a depression that I have been dragging ever since. (COVAR0019)

I don't know if my defences will respond well or not because I'm like this. I suffer from stress. (COVAR0011)

Also, some participants take fewer risks (e.g. avoid climbing stairs) for fear of injuring themselves and having to go to the hospital:

Not being able to do anything and the fear of catching COVID has made me very depressed. For me the worst thing has been, not even being able to see my doctor, that has created a lot of anxiety in me. (COVAR0013)

Before I used to climb a ladder, but now it scares me, because I'm scared of falling and having to go to the hospital. I feel like a deflated balloon now. (COVAR0020)

### 3.4 Theme 4: Vulnerability of testing positive for COVID‐19 due to rheumatoid arthritis

Some participants felt more vulnerable to COVID‐19 due to having rheumatoid arthritis, since the treatment of their own condition weakens the immune system.

I have always been told that my defences go down with the treatments. (COVAR0001)

I was very scared because of my illness because we take so many things … The medications are very strong. (COVAR0002)

Those of us who have an autoimmune disease and with treatments that lower our defences don't know what can happen either, and that makes me feel insecure. They told me that I was immunosuppressed, and it scared me a lot. (COVAR0011)

I felt that it could be worse because of the disease I have and the consequences that it could have in the case of catching it scared me. (COVAR0003)

I was very afraid that with my disease they would have to admit me because I know that I have a higher risk than other people. I have felt more vulnerable, that is why I take better care of myself. (COVAR0016)

On the other hand, some participants felt the opposite: by having their immune system compromised, they felt less vulnerable to potential serious complications related to COVID‐19:

At first I thought I was going to have much more risk, but I think it has protected me. (COVAR0008)

At first, I was very scared because we didn't know how the virus behaved, but in general, I haven't felt vulnerable because of my illness. (COVAR0006)

They really do not know if their treatments protect them or not, although some suspect they do.

## 4 DISCUSSION

This qualitative study has achieved its aims of exploring experiences of patients with rheumatoid arthritis in terms of physical activity and health status during and after the COVID‐19‐induced quarantine. It has also revealed information about the social implications of the COVID‐19 pandemic and vulnerability of testing positive for COVID‐19 due to rheumatoid arthritis.

The results of this study make it clear that even though the patients declared that physical activity is essential for people with rheumatoid arthritis to deal with their disease, most of the participants affirmed that they significatively reduced their levels of physical activity during the pandemic. Balchin et al. (2022) concluded in their cross‐sectional study that more people with rheumatoid arthritis reported decreased physical activity than the general population during the COVID‐19 lockdown. Overall, people with rheumatoid arthritis are less physically active and experience more barriers than the general population (Sokka et al., 2008; Veldhuijzen van Zanten et al., 2015), and sadly, the pandemic has exacerbated this inactive behaviour. The authors identified that this inactive habit may have implications for disease outcomes, as most of the participants claimed that their levels of joint pain have increased, as well as the difficulty of practising sport and fatigue. Currently, the problem is that some of the participants find themselves in a vicious circle of inactivity and fatigue. After being inactive for a long period of time, they felt that they were not able to carry out their typical pre‐pandemic activities, suffering from more pain and a general health detriment; consequently, they became more physically inactive, being more difficult to escape from that vicious cycle.

We identified only two participants who claimed that they exercised during lockdown at the same level than pre‐pandemic. Participants affirmed that their physical inactivity may be due to the restrictions, the lack of motivation and the fear of going outside, which made it impossible for them to go to the gym and practise sport with their friends. Also, they declared that they refused to carry out some activities, such as climbing a ladder, due to the fear of injuring themself and having to go to hospital.

The pandemic has affected participants' mental well‐being, due to the lack of outdoor stimulation and social interaction and the fear of testing positive for COVID‐19. Some of them even declared that they have been diagnosed with depression. It is important to highlight that previous studies reported that people with rheumatoid arthritis present difficulty managing negative emotions (Michaud et al., 2020). Brady et al. (2021) concluded that physical activity is associated with psychological well‐being and mental health and in people with rheumatoid arthritis during COVID‐19, showing a positive relationship and suggesting that maintaining physically active is necessary for maintaining mental functioning, meaning that physical activity counteracts depressive symptoms in people with rheumatoid arthritis. Therefore, this could explain our results related to the association between psychological affects and the reduction in physical activity.

Consequently, it can be shown that the sedentary behaviour that participants claimed has made an impact on the participants quality of life. As Lévy‐Weil et al. (2021) concluded in their study including participants with rheumatoid arthritis, the more physical active the patients continued doing during the pandemic, the greater the positive impact was on quality of life.

The vulnerability of testing positive for COVID‐19 due to rheumatoid arthritis remained uncertain for the included participants. Some of the participants believed that they were under a greater risk of testing positive because their condition is commonly managed by immunosuppressive therapies. On the other hand, a minority of the participants believed that as their immune system is depressed, their treatments strengthen it; hence, they believe that they are more protected and less likely to test positive than the general population. Recent studies concluded that, regardless of preliminary evidence suggesting that infection risk was not increased in rheumatoid arthritis (Figueroa‐Parra et al., 2020), the majority of people with rheumatoid arthritis are under immunosuppressive treatments, which means that they present with an increased risk for compromising their immune response and presenting infections due to COVID‐19 (van Zanten et al., 2020).

This study has important strengths since it explores experiences of people with rheumatoid arthritis in terms of pain and foot health during the pandemic period. Also, this study could be the baseline for the nurse manager's role in educating staff, patients and family. This concept is pointed out in the following fourth recommendation by EULAR: ‘Nurses should participate in the comprehensive management of the disease to control activity and reduce symptoms to improve established patient outcomes’ (van Eijk‐Hustings et al., 2012). Greater involvement of rheumatology nurses in the provision of comprehensive patient care may improve outcomes of disease activity and quality of life related to health (Ndosi et al., 2014). Therefore, it faithfully represents the sensations perceived by patients in situ and not as a memory of that period. However, this study has some limitations to highlight. Firstly, the sample is composed mostly of women, since rheumatoid arthritis has a higher prevalence in this gender. However, this may have influenced Theme 4, ‘Vulnerability of testing positive for COVID‐19 due to rheumatoid arthritis’. This vulnerability could have been influenced by the feeling of family burden, driven by a culture that adds more pressure on women to take care of their families. Secondly, previous studies have concluded that people with rheumatoid arthritis who are treated with biologics, reduce their disease outcomes and can reach remission (Gono et al., 2010). But the present study brings to light that the effectiveness of disease‐modifying anti‐rheumatic drugs (DMARDs) may be influenced by inactivity periods, as the included participants experienced stiffness, pain and loss of functionality after a long time without activity. This highlights again the importance of physical activity as a coadjuvant treatment.

Future work should aim to study the effectiveness of biologics considering the prolonged periods of inactivity in a more homogeneous sample. That future work should be studied through long‐term quantitative studies where the real effectiveness of the treatment can be determined and the experiences of patients in terms of physical activity and foot health can be explored.

## 5 CONCLUSIONS

The present study has revealed that physical activity should be promoted in all people with rheumatoid arthritis, even in difficult times, such as a pandemic, to improve disease outcomes, wellbeing and mental health, in spite of functional disability.

## 6 IMPLICATIONS FOR NURSING MANAGEMENT

Knowing the experiences of these patients enable nursing managers to develop interventions that ensure the delivery of comprehensive nursing care in terms of physical activity and health status, in future situations like this pandemic. Like other nursing specialties, patients with rheumatoid arthritis are highly benefited from the progress in this area. This progress may be due to multidisciplinary teamwork, where nurses play a relevant role, leading and heading projects, giving their point of view in important situations such as the isolation management and restriction on patients' physical and social activity and its consequences, which have been demonstrated after an isolation progress due to the pandemic, and it also has led to kinesiophobia (Minnock et al., 2018).

## CONFLICT OF INTEREST

The authors declare no conflict of interest.

## ETHICS STATEMENT

This qualitative study received ethical approval from the committee of Portal de Ética de la Investigación Biomédica de Andalucía (PEIBA) (SPAR‐001), which was authorized and extended to a longitudinal study ARC0001. This study was carried out in full accordance with the provisions of the Declaration of Helsinki regarding ethical principles for medical research involving human subjects and was approved by the Ethics Committee. All participants in the study provided informed and written consent.

## AUTHOR CONTRIBUTIONS

LRP, ARC and JGC have made substantial contributions to conception and design, acquisition of data, analysis and interpretation of data. GGN, ABOA and GB have been involved in drafting the manuscript and revising it critically for important intellectual content. All authors have read and approved the final version of the manuscript. Each author should have participated sufficiently in the work to take public responsibility for appropriate portions of the content. All authors agreed to be accountable for all aspects of the work in ensuring that questions related to the accuracy or integrity of any part of the work are appropriately investigated and resolved.

## Data Availability

The data that support the findings of this study are available from the corresponding author upon reasonable request.
